# Delays in Achieving Maternal, Newborn, and Child Health Targets for 2021 and 2030 in Liberia

**DOI:** 10.3389/fpubh.2019.00386

**Published:** 2019-12-13

**Authors:** Ulrich Laaser, Raphael Broniatowski, Stephen Byepu, Vesna Bjegovic-Mikanovic

**Affiliations:** ^1^Section of International Public Health, School of Public Health, Bielefeld University, Bielefeld, Germany; ^2^EPOS Health Management, Bad Homburg, Germany; ^3^College of Business Administration, INHA University, Incheon, South Korea; ^4^Faculty of Medicine, School of Public Health and Management, University of Belgrade, Belgrade, Serbia

**Keywords:** gap analysis, delays in progress, health targets, maternal health, newborn health, child health, Liberia

## Abstract

**Objectives:** The Government of Liberia has set ambitious national health targets for 2021 to reduce the high maternal, newborn, and child mortality rate and to improve the related health services. Additionally, Sustainable Development Goal 3 provides a long-term target for 2030. The objective of this article is to analyze the gaps between the targets and collected data.

**Materials and Methods:** Relevant national documents were scrutinized to identify targets and related indicators which can serve as benchmarks for future achievements in Liberia's maternal, newborn, and child health. For each indicator, progress observed will be compared with that needed to meet the target, based on the indicator value in a baseline year, a later observed value, and the expected value in 2021 and 2030, respectively.

**Results:** The Gap Analysis reveals achievements and serious delays for 21 health and health system indicators. Based on national data the reduction of the maternal mortality ratio will take an additional −8.2 years for the 2021 target and −12.5 years for the 2030 target. The Neonatal Mortality rate is experiencing similar delays of −7.9 years for 2021 and −12.9 for 2030 whereas the targets for the Under-5-Mortality rate can be achieved with small delays of −1.8 and −1.7 years.

**Conclusions:** The Government of Liberia requires persistent efforts and international support to achieve its national targets and the Sustainable Development Goal 3 for health.

## Introduction

Liberia, a small Anglophone coastal country in West-Africa, to mean “Land of the Free,” was founded by freed American slaves who were sponsored to settle in Africa as early as 1822. Annexation of land from the indigenous tribes enabled the country to be formed until statehood was declared 1847. The population is comprised of the descendants of the immigration from the United States and of 17 major tribal affiliations, the majority being Christians, a minority of about one-tenth are Muslims. The lack of full integration of the indigenes was the main trigger for the civil war beginning on Christmas Eve in 1989 and lasting until 2003. The enduring confrontation of up to 8 military factions generated a death toll of about 18% of the population of 4.5 million and nearly one million displaced persons (1, 2). The disabled former combatants begging on the urban streets today tell a moving life experience “in their own words” (3).

Based on the last available Census (4) in 2008, about 47% Liberians live in urban areas. Half of the population is below 19 years of age and the dependency ratio is as high as 83%. The Government of Liberia (GoL) has set ambitious national targets for 2021 to reduce the high maternal mortality ratio and to provide and improve the corresponding health services and their determinants as provided by the GoL (5). Since their adoption at the end of 2016, the Sustainable Development Goals (SDGs), especially Goal 3 devoted to health, provide a long-term perspective, focusing on targets set for 2030 (6, 7).

After the decade of civil war the Liberian Gross Domestic Product (GDP) experienced a steep decline in real terms from its peak in 1989 of 1,217 USD per capita to only 247 USD in 2010 with a slight recovery to 454 USD in 2013. In 2014 the improvement of living standards was halted by the Ebola Virus Disease (EVD) crisis which saw facility based deliveries decreased by 30% or more—even in less affected counties [e.g., (8)]. It also contributed to another reduction of GDP per capita (−3.1%). For the fiscal year 2014/15 the GoL allocated only 12.4% or 63 million USD of its total budget to health thereby missing the target of 15% of the national budget as fixed in the Declaration of Abuja in 2001 (9) where the African Union countries met and agreed on an increase in health funding. Of the country's health staff about two out of five are not on the payroll of the GoL and almost 1/3 of the population lives more than 5 km from the nearest health facility (5). In such circumstances all determinants of health, particularly Maternal, Newborn, and Child Health (MNCH) is jeopardized.

The public health work force on payroll, during 2014/15, included only 117 physicians, 436 physician assistants, 2,137 nurses, and 659 midwives (1.2 per 10,000 population), the figures varying significantly between counties in Liberia (10). These numbers are far below the necessary levels proposed by the World Health Organization (WHO) to avoid a critical shortage: According to their guidelines 23 health workers per 10,000 are considered as necessary to secure essential maternal and child health services to the entire population (11, 12): In Liberia there are only 11.8 per 10,000 population, well-short of the guidelines.

Maternal, Newborn, and Child Health (MNCH) are interconnected and related to care for the reproductive health of women and their off-spring. It needs to address all developmental stages: infancy, childhood, adolescence, reproductive, and post-reproductive periods. In order to advance the most important dimensions of universal health coverage i.e., availability, accessibility, acceptability, and quality, in resource limited countries (13) such as Liberia, external support is essential. MNCH requires unrestricted access to care provided by families, communities, outpatient and outreach services, and to clinical services (2, 14, 15).

The purpose of the Liberian policy actions in the health sector are described as building a resilient health system through: (a) improved access to safe and quality health services, (b) health emergency risk management, and (c) enabling social environment and restoring trust. Liberian policy makers set eight targets, and eight aligned indicators for the successful monitoring of activities related to the policy actions (5). By conducting a thorough Gap Analysis, policy makers can analyze the actual situation and resources available, against the targets set. They can then identify what additional resources are needed from local, national, and international agencies in order to support any deficient areas, influencing the targets with the goal of narrowing the gap between the observed situation and the targeted future.

Recognizing the particular challenges facing MNCH care in Liberia, this analysis focuses on key achievements and capacity-gaps related to the implementation of main policies (2), and aims to promote a broader engagement of all stakeholders (national and international partners) to agree on specific recommendations. To this end, the targets set in national documents, especially the Investment Plan for Building a Resilient Health System 2015–2021 (5), as well as international agendas especially the Sustainable Development Goals (SDGs) and the Global Strategy for Women's, Children's and Adolescents' Health (2016–2030) will serve as benchmarks for future achievements in Liberia's MNCH. The framework for SDG monitoring includes 27 indicators for the monitoring of SDG-3 (“Ensure healthy lives and promote well-being for all at all ages”), out of which a group of 16 indicators is directly related to health status (16).

## Materials and Methods

The Gap Analysis follows the structure of the Liberian Investment Plan for Building Resilient Health System (2015–2021) (5): the “main goal, purpose, and outputs per investment area.” In addition to the national targets, we also analyzed indicators relating to SDG-3 targets published in 2017 (16). Comprehensive calculations of all gaps are presented in Annex 2. To calculate the gaps it was essential to have available baseline and observed values—at a later point in time—for particular years. Baseline values for all indicators are those, which were recorded in 2007 and published in the National Health and Social Welfare Policy and Plan 2011–2021 (17). Observed values are the last available and reported in the national health survey in 2013 (18). In addition, point estimates for 2015, published by WHO in 2016 (19), are considered as observed values when available.

For each indicator, progress observed will be compared with that needed to meet the targets (assuming a linear progression). The time gap for an individual indicator will be calculated as the difference between the time remaining to the target year and the time needed to achieve the target (assuming the speed foreseen by the national agenda in Liberia).The time gap (G) is determined in the following way [method based on UNDP in assessing progress toward Millennium Development Goals (MDGs) (20)]:

$$\begin{array}{l} \text{G}={\text{T}}_{\text{r}}-{\text{T}}_{\text{n}} \end{array}$$

Where, T_r_–remaining time is:

$$\begin{array}{l} {\text{T}}_{\text{r}}={\text{t}}_{\text{t}}-\;{\text{t}}_{\text{c}} \end{array}$$

t_t_ and t_c_ denote the target year and the year of observation, respectively.

T_n_–time needed to achieve the target (assuming linear progress) is:

$$\begin{array}{l} {\text{T}}_{\text{n}}={\text{t}}_{\text{t}}-[{\text{t}}_{\text{b}}+{\left({\text{t}}_{\text{t}}-{\text{t}}_{\text{b}}\right)}^{*}({\text{x}}_{\text{c}}-{\text{x}}_{\text{b}})\text{/}({\text{x}}_{\text{t}}-\;{\text{x}}_{\text{b}})] \end{array}$$

t_b_–baseline year,

x_b_–baseline value of the indicator,

x_t_–target value of the indicator,

x_c_–observed value of the indicator.

Positive values of the time gap reflect over-performance i.e., being on track, negative values indicate underperformance (i.e., time lag).

Based on this target-by-target analysis, the report then identifies objectives that are “on track,” “likely,” or “unlikely” to achieve the national target.

The borderline for “unlikely” has been set at a (negative) time gap Gq being more than one quarter of the remaining time:

Gq = G / Tr <-0.25.

## Results

In Table 1 the analysis refers to the main goal—measuring achievements in MNCH mortality. All indicators have values for the national targets to be accomplished up to the year 2021.

**Table 1:** Tracking the main goals of Liberian health policy set by national targets.

**Goal/ Target/ Indicator**	**Baseline value**	**National target 2021**	**Observed value**	**Assessment**	
				**Gap in years up to 2021**	**Performance**
Maternal mortality ratio per 100,000 live-births	994	497	^*^1072	−8.2	Unlikely
			^**^725	−0.4	Likely
Neonatal mortality rate per 1,000 live-births	32	19	^*^38	−12.5	Unlikely
			^**^24	−8.4	Unlikely
Infant mortality rate per 1,000 live-births	71	22	^*^54	−1.1	Likely
Under-5 mortality rate 1,000 per live-births	110	57	^*^94	−1.8	Likely
			^**^70	+2.6	On track

* National health survey in 2013 (18).

** *WHO point estimate for 2015, published in 2016 (19)*.

Based on the national data and envisioned national targets (presented in Table 1), Liberia is projected to reach the target for infant mortality set at 22 infant deaths per 1,000 live-births only one year later than planned. For the national target of 497 maternal deaths per 100,000, the national data and the WHO data provide starkly different projections. The 2013 national health survey data predicts that Liberia will need an additional 8 years to achieve the desired target, while the 2015 WHO estimates predict the national target will be achieved with less than half year delay. The target for neonatal mortality of 19 deaths per 1,000 live-births, is predicted to be severely delayed by both national data and the WHO data with the data sets predicting 12 and 8 years of delay, respectively. The projection is more encouraging for under-5 mortality. Values observed in the 2013 national health survey predict it will be reached in 2023, while the 2015 WHO data projects the national target will be achieved in 2018 (Table 1). For a summary table with all details see Annex 1 and for the detailed calculations Annex 2.

Assuming that the benchmark values are those set by the SDG (Table 2) and all indicators will follow linear trends up to 2030, the latest published values by WHO indicate that Liberia is on track for all targets related to the main goal of its health policy).

**Table 2:** Tracking the main goals of Liberian health policy set by SDGs targets.

**Goal/ Target/ Indicator**	**Baseline value**	**SDG** **target** **2030**	**Observed value**	**Assessment**	
				**Gap in years up to 2030**	**Performance**
Maternal mortality ratio per 100,000 live-births	994	70	^*^1072	−7.9	Unlikely
			^**^725	+0.04	On track
Neonatal mortality rate per 1,000 live-births	32	12	^*^38	−12.9	Unlikely
			^**^24	+1.2	On track
Infant mortality rate per 1,000 live-births	71	Not available	^***^54		Not possible to analyze
Under-5 mortality rate per 1,000 live-births	110	25	^*^94	−1.7	Likely
			^**^70	+2.8	On track

* National health survey in 2013 (18).

** WHO point estimate for 2015, published in 2016 (19).

*** *Among indicators for measuring progress in achieving SDG-3 infant mortality rate is not considered*.

The projections are much different when we consider the 2013 nationally observed values for maternal and neonatal mortality. According to the Gap Analysis Liberia will reach the SDG target for maternal mortality of 70 per 100,000 live births in 2038, delay of 7.9 years. The situation is even worse for neonatal mortality, as it is projected Liberia will reach the SDG target of 12 neonatal deaths per 1,000 live births only in 2043.

Table 3 presents the Gap Analysis of access to safe and quality services, health emergency risk management, and restoring an environment of trust. Liberian policy makers have rightfully given high importance to the monitoring of those indicators taking into account the significant national and donor investment in the health care delivery system. Out of 8 indicators for monitoring the progress toward targets [as published in the Investment Plan, page 35 (5)] and related to maternal and newborn health this analysis could not include “Couple-year protection with family planning methods” because a national target had not been set. Nevertheless, due to the importance of family planning in the Gap Analysis we added the indicator, which is proposed in the SDGs' agenda: “Percentage of women 15–49 years who are sexually active and their need for family planning satisfied with modern methods.”

**Table 3:** Tracking the objectives of Liberian health policy set by national targets for building a resilient health system for women and children.

**Target/ Indicator**	**Baseline value**	**National target 2021**	**Observed value 2016**	**Assessment**	
				**Gap in years up to 2021**	**Performance**
Percentage of children under 1 year who are fully immunized	65	91	60	−4.5	Unlikely
Percentage of children under 1 year who received DPT 3/Penta-3 vaccination	74	91	65	−11.8	Unlikely
Percentage of pregnant women attending 4 ANC visits	54.4	85	58	−0.9	Likely
Percentage of deliveries: facility based and attended by skilled personnel	22	80	51	+1.4	On track
Percentage of pregnant women provided with 2nd dose of IPT-2 for malaria	29	80	41	−3.4	Unlikely
Percentage of HIV+ pregnant women who received antiretroviral treatment	42	80	54	−0.5	Likely
Couple-years protection with family planning methods	45,8	Not available	71,7	Not possible to analyze	Not possible to analyze
% women 15–49 years who are sexually active and their need for family planning satisfied with modern methods	41.6	60	^*^37	−4,0	Unlikely

* *WHO point estimate for 2015, published in 2016 (19)*.

Though time is necessary to observe progress, some targets are lagging behind. The worst situation concerns the target related to immunization of children measured by Universal Health Coverage (UHC) tracer indicators: the perceived gap in achieving 91% of children under 1 year who received DPT3/Penta-3 (Diphtheria, Pertussis, Tetanus) vaccination is close to 12 years, meaning that Liberia will only reach the national target in 2033 instead of 2021. In contrast, maternal health services are doing much better. Liberia is fully on track to achieve the national target in 2021 set for “Facility based and attended by skilled personnel deliveries.” The national target of 4 antenatal care visits (ANC) by 85% of pregnant women in 2022 is delayed only by 0.9 years.

Table 4 presents the results of the Gap Analysis for 3 indicators, provided in international documents to monitor SDGs agenda (11) with defined values of global targets for 2030: (1) percentage of pregnant women provided with 2nd dose of Intermittent Preventive Treatment (IPT-2) for malaria, (2) percentage of (Human Immunodeficiency Virus (HIV) positive pregnant women who received antiretroviral treatment, and (3) share of women 15–49 years who are sexually active and their need for family planning is satisfied with modern methods. With consideration of targets as set by the SGD agenda, all indicators are set at full coverage in 2030.

**Table 4:** Tracking the objectives of Liberian health policy for building a resilient health system for women and children set by SDGs targets.

**Target/ Indicator**	**Baseline value**	**SDG** **target** **2030**	**Observed value 2016**	**Assessment**	
				**Gap in years up to 2030**	**Performance**
Percentage of pregnant women provided with 2nd dose of IPT for malaria	29	100	41	−2.6	Likely
Percentage of HIV+ pregnant women who received antiretroviral treatment	42	100	54	+0.5	On track
% women 15–49 years who are sexually active and their need for family planning satisfied with modern methods	41.6	100	^*^37	−3.3	Unlikely

* *WHO point estimate for 2015, published in 2016 (19)*.

Liberia is on track to achieve the full coverage of all HIV positive pregnant women with antiretroviral treatment within the targeted timeframe. However, full coverage of the same population with a 2nd dose of IPT for malaria, following the SDG target, is projected to require an additional 2.6 years. The target that 100% of reproductive women (in the age group 15–49 years) who are sexually active, will have their needs for family planning satisfied with modern methods, is unlikely to be achieved in time as the calculated delay is −3.3 years (Table 4).

The Gap Analysis encompasses baseline, observed, and targeted values of indicators directly or indirectly related to outputs per investment area of MNCH. The results are presented in Table 5. Based on available data, the calculations of gaps were possible for indicators of the following six out of nine investment areas: Health workforce, Health infrastructure, Medical supplies and diagnostics, Quality service delivery, Information and communication management, and Health financing systems.

**Table 5:** Tracking the outputs per investment area of Liberian health policy set by national targets.

**Targets/Indicator**	**Baseline value (varying; for details see Annex 1**	**National target 2021**	**Observed value 2016**	**Assessment**	
				**Gap in years up to 2021**	**Performance**
**1. INVESTMENT AREA: HEALTH WORKFORCE**					
Core health workforce (physicians, nurses, midwives, physician assistants) per 10,000 population	5.7	14.0	11.4	+1.6	On track
**2. INVESTMENT AREA: HEALTH INFRASTRUCTURE**					
Percentage of population living within 5 km from the nearest health facility	69	85	71	−1.6	Likely
Health facility density per 10,000 population	1.6	2.0	1.9	+3.5	On track
Percentage of health facilities with all utilities ready to provide services (water, electricity)	55	100	59	−4.5	Unlikely
**4. INVESTMENT AREA: MEDICAL SUPPLIES and DIAGNOSTICS**					
Percentage of facilities with no stock-out of tracer drugs during the period (amoxicillin, cotrimoxazole, paracetamol, Oral Rehydration Salts (ORS), iron folate, ACT, FP commodity)	62.3	95	44	−12.2	Unlikely
**5. INVESTMENT AREA: QUALITY SERVICE DELIVERY**					
Number of blood units collected	836	100,000	Not available	Not possible to analyze	Not possible to analyze
Percentage of facilities reaching two stars level in accreditation survey, including clinical standards	9.3	90	Not available	Not possible to analyze	Not possible to analyze
OPD consultations per inhabitant per year	0.9	2.0	0.7	−8.0	Unlikely
**6. INVESTMENT AREA: INFORMATION and COMMUNICATION MANAGEMENT**					
Percentage of timely, accurate and complete Health Information System (HIS) reports submitted to MoH during the year	36	90	78	+3.2	on track
**9. INVESTMENT AREA: HEALTH FINANCING SYSTEMS**					
Per capita health expenditure per year (USD)	65.0	80.0	64	−3.5	unlikely
Public expenditure in health as % of total public expenditure	7.8	15.0^*^	12.4	+1.0	on track
Out of pocket payment for health as a share of current expenditure on health	51	15	Not available	Not possible to analyze	Not possible to analyze

* *As in the Abuja Agreement (9)*.

Following Universal Health Care (UHC) requests, Liberia is steadily attempting to increase physical access to health services and had a chance to achieve the national target set as a share of 85% of population living within 5 km from the nearest health facility well in time, were it not for the devastating effects of the Ebola Virus Disease (EVD) crisis in 2014/15. The population living within the 5 km range increased between 2010 and 2016 by 2% only (see also Annex 1) i.e., from 69 to 71%. OPD (Out Patient Department) consultations (per inhabitant and year) even decreased from 0.9 to 0.7 with a resulting delay of 8 years. The functionality of the infrastructure is very limited in regards to water and electricity and availability of drugs (Table 5, investment area 2 and 4).

## Discussion

To the best of our knowledge we present here the first comprehensive gap analysis for a number of selected indicators relating to MNCH and related health services in Liberia. In Table 6 we summarize the Liberian achievements and deficits.

**Table 6:** Summary of target achievements, indicators based on national Liberian data.

**Status of target achievement**	National targets 2021^*^	SDG targets 2030^**^
On track	6	1
Likely	5	2
Unlikely	10	3
**Total**	**21**	**6**
Missing data	5	0

* Tables 1, 3, 5.

** *Tables 2, 4*.

The results of our analysis indicate that based on national data only half of all indicators are likely to be achieved by 2021 and 2030, respectively. This applies especially to maternal and newborn mortality but also to service indicators such as “Percentage of children under 1 year who received DPT3/Penta-3 vaccination” or “Percentage of pregnant women provided with 2nd dose of IPT for malaria.” The situation is more positive for older children, infants and under-fives, who are on track for 2021 as well as for 2030. In comparison to other member states of the Economic Community of West African States (ECOWAS) Liberia predominantly ranks in the upper third although only Capo Verde and Ghana show reasonably positive development over the last years (21). The reasons for such varied levels of achievement in many cases are due to the shortage of vaccines and drugs or availability of competent staff. Maternal waiting homes proposed to have high risk women nearby for the last months of pregnancy (especially if they live in a distance of more than 5 km from the next facility) do not seem to affect this situation. In addition many women cannot leave their household work or work in the fields even toward the end of their pregnancy. However, recent publications see some success regarding the proportion of the targeted women utilizing these homes (22, 23).

### Limitations

Taking into account the abundance of policies addressing MNCH in Liberia, this analysis has faced the difficulty that baseline figures and targets are not always identical for all documents when addressing the same MNCH issues. The assumption of a linear progression calculated from a baseline year to an observed year assuming its straight continuation to the target year is an artificial but neutral one, although subject to multiple modifying factors in a rarely foreseeable future. However, it should stimulate the identification of potential positive impacts and their advancement i.e., either continuing success or increasing efforts to compensate a delay. The reliability and validity of the available data can be questioned as well, e.g., referring to the values for the Maternal Mortality Ratio in Annex 1 where we see a difference of 1072 vs. 725 equal to a difference of 347 per 100,000 live births achieved within 2 years, which is rather unlikely. In addition MNCH deaths are rarely reported if happening at home. While national and international standardization of definitions of indicators, the quality of data, and their availability constitute a limitation for the time being, the next generation of demographic and health surveys with better quality will become available soon (envisioned for 2019). The assumption of achieving full coverage for some operational indicators in 2030 is not realistic. Furthermore, some documents miss a quantitative definition of targets of interventions and therefore process indicators are difficult to evaluate. In general and understandably, considering the time when documents have been endorsed, they follow the MDG framework (24) and do not consider the recent SDGs approach. Therefore, in the future a priority will be to tailor and localize SDGs toward the specific country situation in Liberia.

### Future Policy Development

Both, the SDG framework (13) and the Global Strategy for Women's, Children's and Adolescents' Health (2016–2030) (25) put special attention to the formulation of national health policies. For this purpose United Nations Country Teams (UNCTs) have developed a Reference Guide to support countries in tailoring SDGs (26). In addition, the African Union adopted a Health Strategy, based on SDGs to lead the region from 2016 to 2030 (27). In its National Development Plan (NDP) GoL (28) points to a key requirement for country-wide implementation i.e., partnering with donors, Non-Governmental Organizations (NGOs), and others active in the health sector.

Many determinants, already described in the Investment Plan, contribute to the situation uncovered by this Gap Analysis. Certainly several relevant interventions would be possible to increase qualified service availability by strengthening the health workforce through education and obligatory and controlled Continued Professional Development (CPD) with a continuation of investments into the health system and physical infrastructure (roads, electricity, and Internet). In addition, for advancement in accessibility, promising interventions to develop population health literacy and overall trust of citizens in the health system, preferably through community and health promotion programs are planned.

Liberian policy makers re-considered the revision of desired values in the next health policy plan (for the period after 2021): a national governmental group in cooperation with UNDP has in this context performed an analysis of all SDGs in December 2016 by using a “Rapid Integrated Assessment” tool. Following the advice that each country takes into consideration its own dynamic and unique situations when setting national targets, the group demonstrated that Liberia is an innovative case of good practices where these analyses are informing the mid-term review of the National Development Plan (29). For example the new WHO guidelines on antenatal care (ANC) visits (30) request eight or more whereas the actual guidelines set a target-indicator of four ANCs. Whether a standard of eight ANCs is realistic or not, is a decision for health policy makers in the near future. However, by striving for the label of a middle income country to be achieved by 2030, the Liberian Government is recognizing health as standing in the very center of development (31). Such recognition by the GoL can be considered an excellent base for the Liberian health sector policy makers to efficiently build a resilient health system.

In general, this Gap Analysis in spite of its limitations (especially the data quality and the assumption of linear progression) can become useful for Liberian policy makers who may take advantage of reconsidering the values for the national health targets more realistically by adopting SMART objectives [**S**pecific, **M**easurable, **A**chievable, **R**elevant, **T**ime-Oriented (32)] and looking for international benchmarks. To that end the Liberian Ministry of Health (MoH) monitors progress through the national demographic and health surveys done by the Liberia Institute of Statistics and Geo-Information Services (LISGIS) and occasional assessments initiated by international organizations, such as the SARA reports (Service Availability and Readiness Assessment and Quality of Care Report) (33).

### Conclusions and Recommendations

The newly established National Institute of Public Health together with the Liberia Institute of Statistics and Geo-Information Services (LISGIS) has been ordered by the Ministry of Health (MoH) to create a database with complete and up to date coverage of all health indicators serving to monitor the main goals, purpose, and investment areas of health policy with a special focus on the SDGs.

The registration of births and deaths according to the codes of the International Classification of Diseases (ICD) is one of the priorities and can be improved by well-trained and controlled coding staff especially regarding still-births, neonatal and post-neonatal period, and for deaths together with their causes in reproductive women, children, and adolescents. The National Institute of Public Health together with the LISGIS have the ability to provide improved statistics by the end of 2021 to the MoH, given sufficient international support and resources.

Likewise the MoH may request the same two institutions to develop databases with fully disaggregated health data publicly accessible free of charge by 2021.

The Liberian health policy makers may consider changing several indicators for the monitoring of certain achievements. In family planning, instead of “Number of Total Couple Year Protection (all methods),” according to methods supporting an UHC approach of WHO, it should be worded: “Proportion of married or in union women of reproductive age who have their need for family planning satisfied with modern methods.” Instead of “fully immunized infants,” the tracer indicator for Universal Health Coverage (UHC) is “DTP3 immunization coverage among 1-year olds.” Instead of “TB detection rate” Liberian health policy makers could decide to follow up on “utilization of TB treatment.” Reconsidering the exact definition and wording of key indicators would increase reliability and relevance of findings.

Besides already utilized surveys, such as the Liberian Demographic and Health Survey and SARA (the annual monitoring system for service delivery), Liberia can also use other surveys important for monitoring and evaluation of MNCH, and supported by international organizations. This recommendation particularly refers to MICS (Multiple Indicator Cluster Survey, organized and usually funded by the United Nations International Children's Emergency Fund (UNICEF).

Special attention has to be paid to increase the coverage by mother and child health services including exact and complete registration. Guidelines comprising monitoring and interventions to that aim can be developed by the National Institute of Public Health together with the maternal and pediatric services until 2030.

As there is considerable unmet need for birth control together with a high rate of early pregnancies in Liberia, the community health services are supported by the Ministry of Health (MoH) to make special efforts on individual interventions in this area and to report on their success each year.

Liberian health professionals and decision makers have an opportunity to follow up on the recently published advancement in MNCH related science, such as those which present estimates of the costs and benefits of progressively expanding health services in order to reach SDGs health targets in low- and middle-income countries. This approach has the potential to strengthen requests for funding by the international community.

In Summary, according to the last step of the policy cycle i.e., monitoring and evaluation a comprehensive efficiency analysis of the scarce resources available may lead to a modification of priorities focusing on data quality i.e., reliability and validity of core or tracer indicators adopted at the global level for Universal Health Coverage and the Sustainable Development Goals to enable a permanent update of relevant information for policy making and adjustment.

## Author Contributions

UL integrated the contributions of the co-authors and wrote the paper. RB assembled the data. SB calculated the statistical parameters. VB-M conceptualized the study.

### Conflict of Interest

The authors declare that the research was conducted in the absence of any commercial or financial relationships that could be construed as a potential conflict of interest.
