# Supplementary material for: Delays in Achieving Maternal, Newborn, and Child Health Targets for 2021 and 2030 in Liberia
Source: Front Public Health. 2019 Dec 13;7:386. doi: 10.3389/fpubh.2019.00386 (PMC6923216; doi:10.3389/fpubh.2019.00386)
Supplement: Supplementary file 1 [file Data_Sheet_1.docx]

**Annex 1: Gap Analysis for National and International (SDG) Targets – 2021 and 2030**

Gap Analysis for National Targets – 2021 and Global Targets – 2030

Indicators taken from the National Plan 2011-2021 (page 83) and the Investment Plan 2015-2021 (page 35).

Methodology taken from the UNDP Regional Bureau for Europe and the Commonwealth of Independent States (2006): National Millennium Development Goals: A framework for action. ISBN: 92−95042−49−2. The borderline for “unlikely” has been set at a time gap lower than one quarter of the remaining time (Gq = G / Tr <-0.25).

| **Goal/ Target/ Indicator** | **Baseline** | | **IP Target 2021** | **SDG Target 2030** | **Observed** | | **Assessment** | | | |
| --- | --- | --- | --- | --- | --- | --- | --- | --- | --- | --- |
|  | **Year** | **Value** |  |  | **Year**** | **Value** | **Gap**  **(yrs) 2021** | **Performance**  **Gq =** *G* */ Tr* | **Gaps (yrs)**  **2030** | **Performance**  **Gq =** *G* */ Tr* |
| **Indicators for monitoring the main goal –Improved health status of Liberian mothers and children**  (these indicators are not exclusive of the health sector and should be measured every 5 years) | | | | | | | | | | |
| Maternal mortality ratio per 100,000 live-births | 2007 | 994 | 497 | 70 | 2013  2015 | 1072  725*** | **-8.2**  **-0.4** | **Unlikely**  **(-1.03)**  **Likely**  **(-0.067)** | **-7.9**  **+0.04** | **Unlikely**  **(-0.47)**  **on track**  **(+0.002)** |
| Neonatal mortality rate per 1,000 live-births | 2007 | 32 | 19 | 12 | 2013  2015 | 38  24*** | **-12.5**  **-8.4** | **Unlikely**  **(-1.56)**  **Unlikely**  **(-1.4)** | **-12.9**  **+1.2** | **Unlikely**  **(-0.76)**  **on track**  **(+0.08)** |
| Infant mortality rate per 1,000 live-births | 2007 | 71 | 22 |  | 2013 | 54 | **-1.1** | **Likely**  **(-0.14)** |  |  |
| Under-5 mortality rate per 1,000 live-births | 2007 | 110 | 57 | 25 | 2013  2015 | 94  70*** | **-1.8**  **+2.6** | **Likely**  **(-0.23)**  **on track**  **(+0.43)** | **-1.7**  **+2.8** | **Likely**  **(-0.1)**  **on track**  **(+0.19)** |

| **Goal/ Target/ Indicator** | **Baseline** | | **IP Target 2021** | **SDG Target 2030** | **Observed** | | **Assessment** | | | |
| --- | --- | --- | --- | --- | --- | --- | --- | --- | --- | --- |
|  | **Year** | **Value** |  |  | **Year** | **Value** | **Gap**  **(yrs)**  **2021** | **Performance** | **Gap**  **(yrs)**  **2030** | **Performance** |
| **Indicators for monitoring the purpose of the Liberian health policy**  (to be monitored every 1-3 years as specific for the health system) | | | | | | | | | | |
| Percentage of children under 1 year who are fully immunized | 2013 | 65 | 91 |  | 2016 | 60 | **-4.5** | **Unlikely**  **(-0.9)** |  |  |
| Percentage of children under 1 year who received DPT3/Penta-3 vaccination | 2010 | 74 | 91 |  | 2016 | 65 | **-11.8** | **Unlikely**  **(-2.4)** |  |  |
| Percentage of pregnant women attending 4 ANC visits | 2013 | 54.4 | 85 |  | 2016 | 58.0 | **-0.9** | **Likely**  **(-0.11)** |  |  |
| Percentage of deliveries: facility based and attended by skilled personnel | 2010 | 22 | 80 |  | 2016 | 51 | **+1.4** | **On track**  **(+0.28)** |  |  |
| Number of skilled birth attendants (physicians,  nurses, midwives and physician assistants) per  10,000 population | 2010 | 5.7 | 14 |  | 2015 | 8.6 | **-1.2** | **Likely**  **(-0.2)** |  |  |
| Percentage of pregnant women provided with  2nd dose of IPT for malaria | 2010 | 29 | 80 | 100** | 2016 | 41 | **-3.4** | **Unlikely**  **(-0.68)** | **-2.6** | **Likely**  **(-0.19)** |
| Percentage of HIV+ pregnant women who received antiretroviral treatment | 2013 | 42 | 80 | 100** | 2016 | 54 | **-0.5** | **Likely**  **(-0.1)** | **+0.5** | **On track**  **(+0.037)** |
| Couple-years protection with family planning methods | 2010 | 45,8 | NA |  | 2013 | 71,7 |  |  |  |  |

| **Goal/ Target/ Indicator** | **Baseline** | | **IP Target 2021** | **SDG Target 2030** | **Observed** | | **Assessment** | | | |
| --- | --- | --- | --- | --- | --- | --- | --- | --- | --- | --- |
|  | **Year** | **Value** |  |  | **Year** | **Value** | **Gap**  **(yrs)**  **2021** | **Performance** | **Gap**  **(yrs)**  **2030** | **Performance** |
| % women 15-49 years who are sexually active and their need for FP satisfied with modern methods | 2013 | 41.6 | 60 | 100** | 2015 | 37*** | **-4.0** | **Unlikely**  **(-0.67)** | **-3.3** | **likely**  **(-0.22)** |
| TB case detection rate | 2008 | 53 | 85 |  | 2015 | 56 | **+1.2** | **on track**  **(+0.09)** |  |  |
| **Goal/ Target/ Indicator** | **Baseline** | | **IP Target 2021** | **SDG Target 2030** | **Observed** | | **Assessment** | | | |
|  | **Year** | **Value** |  |  | **Year** | **Value** | **Gap**  **(yrs)**  **2021** | **Performance** | **Gap**  **(yrs)**  **2030** | **Performance** |
| **Selected indictors directly and indirectly related to MNH for monitoring of outputs per nine investment areas, particularly health system performance**  (these indicators, to be monitored annually, focus on the system’s components and their performance; most should be used also at County level) | | | | | | | | | | |
| **1. Investment area: Health workforce** | | | | | | | | | | |
| Core health workforce (physicians, nurses, midwives, physician assistants) per 10,000 population | 2010 | 5.7 | 14.0 | 44.5  23.5**** | 2016 | 11.4 | **1.6** | **On track**  **(+0.32)** | **-3.1**  **0.4** | **Likely**  **(-0.22)**  **On track**  **(0.03)** |
| **2. Investment area: Health infrastructure** | | | | | | | | | | |
| Percentage of population living within 5 km from the  nearest health facility | 2010 | 69.0 | 85.0 |  | 2013 | 71.0 | **-1.6** | **Likely**  **(-0.20)** |  |  |
| Health facility density per 10,000 population | 2015 | 1.6 | 2.0 |  | 2016 | 1.9 | **3.5** | **on track**  **(+0.7)** |  |  |
| Percentage of health facilities with all utilities ready to provide services  (with water, electricity) | 2015 | 55 | 100 |  | 2016 | 59 | **-4.5** | **Unlikely**  **(-0.75)** |  |  |

| **4. Investment area: Medical supplies and diagnostics** | | | | | | | | | | |
| --- | --- | --- | --- | --- | --- | --- | --- | --- | --- | --- |
| Percentage of facilities with no stock-out of tracer drugs during the period (amoxicillin, cotrimoxazole, paracetamol, ORS, iron folate, ACT, FP commodity) | 2011 | 62.3 | 95 |  | 2016 | 44 | **-12,2** | **Unlikely**  **(-2.4)** |  |  |
| **5. Investment area: Quality service delivery** | | | | | | | | | | |
| Number of blood units collected | 2013 | 836 | 100,000 |  |  |  |  |  |  |  |
| Percentage of facilities reaching two star level in accreditation survey, including clinical standards | 2011 | 9.3 | 90 |  |  |  |  |  |  |  |
| OPD consultations per inhabitant per year | 2010 | 0.9 | 2.0 | 5.0**** | 2016 | 0.7 | **-8.0** | **Unlikely**  **(-1.6)** | **-7.0** | **Unlikely**  **(-0.5)** |
| **6. Investment area: Information and communication management** | | | | | | | | | | |
| Percentage of timely, accurate and complete HIS reports submitted to MOH during the year | 2013 | 36 | 90 |  | 2016 | 78 | **+3.2** | **On track**  **(+0.64)** |  |  |
| **9. Investment area: Health financing systems** | | | | | | | | | | |
| Per capita health expenditure per year (US$) | 2013 | 65.0 | 80.0 |  | 2016 | 64.0 | **-3.5** | **Unlikely**  **(-0.71)** |  |  |
| Public expenditure in health as % of total public expenditure | 2010 | 7.8 | 15.0* |  | 2016 | 12.4 | **1.0** | **on track**  **(+0.2)** |  |  |
| Out of pocket payment for health as a share of current expenditure on health | 2014 | 51 | 15 |  |  |  |  |  |  |  |

***See: Abuja agreement **See: SDG 3.3 ***WHO point estimate for 2015, published in 2016 ****WHO targets assumed for 2030**

**Note: Figures usually are rounded to one decimal point**

**Sources of data:**

| **Years** | **References** | **Websites** |
| --- | --- | --- |
| 2007 | DHS (average of 7 years) | https://dhsprogram.com/pubs/pdf/fr201/fr201.pdf |
| 2008 | National Census | https://www.lisgis.net |
| 2011-2021 | National Health Policy & Plan 2011-2021 for baseline 2010 (MoH), p. 83 | http://moh.gov.lr/2011-2021-national-health-policy-plan-moh/ |
| 2013 | DHS (ave of 7 years) | www.dhsprogram.com/pubs/pdf/FR291/FR291.pdf |
| 2015 | Investment plan 2015-2021 (MoH) | www.humanitarianresponse.info/en/operations/liberia/document/investment-plan-building-resilient-health-system |
| 2016 | Joint Annual Review | http://moh.gov.lr/2016-joint-annual-review-conference-report |
| 2016 | WHO 2017 | World health statistics 2017: monitoring health for the SDGs, Sustainable Development Goals. Geneva: World Health Organization; 2017. Licence: CC BY-NC-SA 3.0 IGO. |

**Annex 2: Calculations**

**MDG Formula:**

*G* time gap

*Tr* remaining time

*Tn* time needed to achieve the target (assuming linear progress)

*tb* baseline year

*tc* year of observation

*tt* target year

*xb* baseline value of the indicator

*xc* observed value of the indicator

*xt* target value of the indicator

Tn= tt − [tb + ((tt – tb) * xc – xb / xt – xb) ] =

Example:

Tn = 2021 – [2010 + ((2021 – 2010) * 78 – 76 / 90 – 76)] =

2021 – [2010 + (11 * 0,14)] = 2021 – [2010 +1,5] = 2021 – 2011,5 = 9,5

Tr = 2021 – 2016 = 5

G = 5 – 9,5 = -4,5 (unlikely)

Gq = -4,5 / 5 = -0,9

**Maternal mortality rate**

**I. Target 2021**

2007 = 0994

2013 = 1072

2021 = 0497

**II. Target 2021**

2007 = 0994

2015 = 0725

2021 = 0497

**III. Target 2030**

2007 = 0994

2013 = 1072

2030 = 0070

**IV. Target 2030**

2007 = 0994

2015 = 0725

2030 = 0070

Ad I.

Tn = 2021 – [2007 + ((2021 – 2007) * 1072 – 994 / 497 – 994)] =

2021 – [2007 + (14 * -0,157)] = 2021 – [2007 – 2,2] = 2021 – 2004,8 = 16,2

Tr = 2021 – 2013 = 8

G = 8 – 16,2 = -8,2 (unlikely)

Gq = -8,2 / 8 = -1.03

Ad II.

Tn = 2021 – [2007 + ((2021 – 2007) * 725 – 994 / 497 – 994)] =

2021 – [2007 + (14 * 0,54)] = 2021 – [2007 + 7,56] = 2021 – 2014,56 = 6,4

Tr = 2021 – 2015 = 6

G = 6 – 6,4 = -0,4 (likely)

Gq = -0,4 / 6 = -0,067

Ad III.

Tn = 2030 – [2007 + ((2030 – 2007) * 1072 – 994 / 70 - 994)] =

2030 – [2007 + (23 * -0,084)] = 2030 – [2007 – 1,94] = 2030 – 2005,06 = 24,94

Tr = 2030 – 2013 = 17

G = 17 – 24,94 = -7,94 (unlikely)

Gq = -7,94 / 17 = -0,47

Ad IV.

Tn = 2030 – [2007 + ((2030 – 2007) * 725 – 994 / 70 - 994)] =

2030 – [2007 + (23 * 0,29)] = 2030 – [2007 + 6,70] = 2030 – 2013,7 = 16,3

Tr = 2030 – 2013 = 17

G = 17 – 16,3 = 0,04 (on track)

Gq= 0,04 / 17 = 0,002

**Neonatal mortality rate**

**I. Target 2021**

2007 = 32

2013 = 38

2021 = 19

**II. Target 2030**

2007 = 32

2013 = 38

2030 = 12

**III. Target 2021**

2007 = 32

2015 = 24

2021 = 19

**IV. Target 2030**

2007 = 32

2015 = 24

2030 = 12

Ad I.

Tn = 2021 – [2007 + ((2021 – 2007) * 38 – 32 / 19 – 32)] =

2021 – [2007 + (14 * -0,46)] = 2021 – [2007 – 6,5] = 2021 – 2000,5 = 20,5

Tr = 2021 – 2013 = 8

G = 8 – 20,5 = -12,5 (unlikely)

Gq = -12,5 / 8 = -1.56

Ad II.

Tn = 2030 – [2007 + ((2030 – 2007) * 38 – 32 / 12 - 32)] =

2030 – [2007 + (23 * -0,3)] = 2030 – [2007 – 6,9] = 2030 – 2000,1 = 29,9

Tr = 2030 – 2013 = 17

G = 17 – 29,9 = -12,9 (unlikely)

Gq = -12,9 / 17 = -0,76

Ad. III

Tn = 2021 – [2007 + ((2021 – 2007) * 24 – 32 / 19 - 32)] =

2021 – [2007 + (14 * +0.62)] = 2030 – [2007 + 8.6] = 2030 – 2015.6 = 14.4

Tr = 2021 – 2015 = 6

G = 6 – 14.4 = -8.4 (unlikely)

Gq = -8.4 / 6 = -1.4

Ad IV

Tn = 2030 – [2007 + ((2030 – 2007) * 24 – 32 / 12 - 32)] =

2030 – [2007 + (23 * +0.4)] = 2030 – [2007 + 9.2] = 2030 – 2016.2 = 13.8

Tr = 2030 – 2015 = 15

G = 15 – 13.8 = +1.2 (on track)

Gq = +1.2 / 15 = 0,08

**Infant mortality rate**

**I. Target 2021**

2007 = 71

2013 = 54

2021 = 22

Ad I.

Tn = 2021 – [2007 + ((2021 – 2007) * 54 – 71 / 22 – 71)] =

2021 – [2007 + (14 * 0,34)] = 2021 – [2007 + 4,9] = 2021 – 2011,9 = 9,1

Tr = 2021 – 2013 = 8

G = 8 – 9,1 = -1,1 (likely)

Gq = -1,1 / 8 = -0,14

**Under-5 mortality rate**

**I. Target 2021**

2007 = 110

2013 = 94

2021 = 57

**II. Target 2021**

2007 = 110

2015 = 70

2021 = 57

**III Target 2030**

2007 = 110

2013 = 94

2030 = 25

**IV. Target 2030**

2007 = 110

2015 = 70

2030 = 25

Ad I.

Tn = 2021 – [2007 + ((2021 – 2007) * 94 – 110 / 57 – 110)] =

2021 – [2007 + (14 * 0,30)] = 2021 – [2007 + 4,2] = 2021 – 2011,2 = 9,8

Tr = 2021 – 2013 = 8

G = 8 – 9,8 = -1,8 (likely)

Gq = -1,8 / 8 = -0,23

Ad II.

Tn = 2021 – [2007 + ((2021 – 2007) * 70 – 110 / 57 – 110)] =

2021 – [2007 + (14 * 0,75)] = 2021 – [2007 + 10.6] = 2021 – 2017.6 = 3,4

Tr = 2021 – 2015 = 6

G = 6 – 3.4 = 2.6 (on track)

Gq = 2.6 / 6 = 0.43

Ad III.

Tn = 2030 – [2007 + ((2030 – 2007) * 94 - 110 / 25 - 110)] =

2030 – [2007 + (23 * 0,19)] = 2030 – [2007 + 4,3] = 2030 – 2011,3 = 18,7

Tr = 2030 – 2013 = 17

G = 17 – 18,7 = -1,7 (likely)

Gq = -1.7 / 17 = -0.1

Ad IV.

Tn = 2030 – [2007 + ((2030 – 2007) * 70 - 110 / 25 - 110)] =

2030 – [2007 + (23 * 0,47)] = 2030 – [2007 + 10.8] = 2030 – 2017.8 = 12.2

Tr = 2030 – 2015 = 15

G = 15 – 12.2 = 2.8 (on track)

Gq = 2.8 / 15 = 0,19

**Percentage of children under 1 year who are fully immunized**

**I. Target 2021**

2013 = 65

2016 = 60

2021 = 91

Ad I.

Tn = 2021 – [2013 + ((2021 – 2013) * 60 – 65 / 91 – 65)] =

2021 – [2013 + (8 * -0,19)] = 2021 – [2013 – 1.54] = 2021 – 2011.46 = 9.5

Tr = 2021 – 2016 = 5

G = 5 – 9.5 = -4.5 (unlikely)

Gq = -4.5 / 5 = -0.9

**Percentage of children under 1 year who received DPT3/Penta-3 vaccination**

**I. Target 2021**

2010 = 74

2016 = 65

2021 = 91

Ad I.

Tn = 2021 – [2010 + ((2021 – 2010) * 65 – 74 / 91 – 74)] =

2021 – [2010 + (11 * -0.52)] = 2021 – [2010 – 5.8] = 2021 – 2004.2 = 16.8

Tr = 2021 – 2016 = 5

G = 5 – 16.8 = -11.8 (unlikely)

Gq = -11.8 / 5 = -2.36

**Percentage of pregnant mothers attending 4 ANC visits**

**I. Target 2021**

2013 = 54,4

2016 = 58.0

2021 = 85,0

Ad I.

Tn = 2021 – [2013 + ((2021 – 2013) * 58.0 - 54,4 / 85 – 54.4)] =

2021 – [2013 + (8 * +0,12)] = 2021 – [2013 + 0.94] = 2021 – 2013,94 = 7.1

Tr = 2021 – 2013 = 8

G = 8 – 7.1 = -0.9 (likely)

Gq = -0.9 / 8 = -0.11

**Percentage of deliveries that are facility-based with a skilled birth attendant**

**I. Target 2021**

2010 = 22

2016 = 51

2021 = 80

Ad I.

Tn = 2021 – [2010 + ((2021 – 2010) * 51 – 22 / 80 – 22)] =

2021 – [2010 + (11 * 0,67)] = 2021 – [2010 + 7.4] = 2021 – 2017.4 = 3.6

Tr = 2021 – 2016 = 5

G = 5 – 3.6 = 1.4 (on track)

Gq = 1.4 / 5 = +0,28

**Number of skilled birth attendants (physicians, nurses, midwives & physician assistants) / 10,000 population**

**I. Target 2021**

2010 = 5,7

2015 = 8,6

2021 = 14

Ad I.

Tn = 2021 – [2010 + ((2021 – 2010) * 8,6 – 5,7 / 14 – 5,7)] =

2021 – [2008 + (11 * 0,35)] = 2021 – [2010 + 3,8] = 2021 – 2013,8 = 7,2

Tr = 2021 – 2015 = 6

G = 6 – 7,2 = -1,2 (likely)

Gq = -1,2 / 6 = -0,2

**Percentage of pregnant women provided with 2nd dose of IPT for malaria**

**I. Target 2021**

2010 = 29

2016 = 41

2021 = 80

**II. Target 2030**

2010 = 29

2016 = 41

2030 = 100

Ad I.

Tn = 2021 – [2010 + ((2021 – 2010) * 41 – 29 / 80 – 29)] =

2021 – [2010 + (11 * 0,24)] = 2021 – [2010 + 2.6] = 2021 – 2012.6= 8.4

Tr = 2021 – 2016 = 5

G = 5 – 8.4 = -3.4 (unlikely)

Gq = -3.4 / 5 = -0,68

Ad II.

Tn = 2030 – [2010 + ((2030 – 2010) * 41 – 29 / 100 – 29)] =

2030 – [2010 + (20 * 0,17)] = 2030 – [2010 + 3.4] = 2030 – 2013,4 = 16,6

Tr = 2030 – 2016 = 14

G = 14 – 16,6 = -2.6 (likely)

Gq = -2.6 / 14 = -0.19

**Percentage of HIV+ pregnant women provided with antiretroviral treatment**

**I. Target 2021**

2013 = 42

2016 = 54

2021 = 80

Ad I.

Tn = 2021 – [2013 + ((2021 – 2013) * 54 – 42 / 80 – 42)] =

2021 – [2013 + (8 * 0,32)] = 2021 – [2013 + 2,5] = 2021 – 2015,5 = 5,5

Tr = 2021 – 2016 = 5

**G = 5 – 5,5 = -0,5 (likely)**

Gq = 0,5 / 5= -0,1

**II. Target 2030**

2013 = 42

2016 = 54

2030 = 100

Ad II.

Tn = 2030 – [2013 + ((2030 – 2013) * 54 – 42 / 100 – 42)] =

2030 – [2013 + (17 * 0,21)] = 2030 – [2013 + 3,52] = 2030 – 2016,52 = 13,48

Tr = 2030 – 2016 = 14

G = 14 – 13,48 = +0,52 (likely)

Gq = 0,52 / 14= 0,037

**% women 15-49 years who are sexually active and their need for FP satisfied with modern methods**

**I. Target 2021**

2013 = 41.6

2015 = 37.0

2021 = 60.0

II. Target 2030

2013 = 41.6

2015 = 37.0

2030 = 100.0

Ad I.

Tn = 2021 – [2013 + ((2021 – 2013) * 37 – 41.6 / 100 – 41.6)] =

2021 – [2013 + (8 * -0,25))] = 2021 – [2013 - 2] = 2021 – 2011 = 10.0

Tr = 2021 – 2015 = 6

G = 6 – 10.0 = -4.0 (unlikely)

Gq = -4 / 6 = -0.67

Ad II.

Tn = 2030 – [2013 + ((2030 – 2013) * 37 – 41.6 / 100 – 41.6)] =

2030 – [2013 + (17 * -0,08))] = 2030 – [2013 – 1.3] = 2030 – 2011.7 = 18.3

Tr = 2030 – 2015 = 15

G = 15 – 18.3 = -3.3 (likely)

Gq = -3.3 / 15 = -0.22

**TB case detection rate (all forms)**

**I. Target 2021**

2008 = 53

2015 = 56

2021 = 85

Ad I.

Tn = 2021 – [2008 + ((2021 – 2008) * 56 – 53 / 85 – 53)] =

2021 – [2008 + (13 * 0,09)] = 2021 – [2008 + 1,2] = 2021 – 2009,2 = 11,8

Tr = 2021 – 2008 = 13

G = 13 – 11,8 = 1,2 (on track)

Gq = 1,2 / 13 = 0,09

**Core Health Work Force (incl. physician assistants) per 10,000 population**

**I. Target 2021**

2010 = 5.7

2016 = 11.4

2021 = 14.0

Tn = 2021 – [2010 + ((2021 – 2010) * 11.4 – 5.7 / 14.0 – 5.7)] =

2021 – [2010 + (11 * 0,69)] = 2021 – [2010 + 7.55] = 2021 – 2017,6 = 3.4

Tr = 2021 – 2016 = 5

G = 5 – 3.4 = +1.6 (on track)

Gq = 1.6 / 5 = +0,32

**II. Target 2030**

2010 = 5.7

2016 = 11.4

2030 = 44.5

Tn = 2030 – [2010 + ((2030 – 2010) * 11.4 – 5.7 / 44.5 – 5.7)] =

2030 – [2010 + (20 * 0,15)] = 2021 – [2010 + 2.9] = 2030 – 2012,9 = 17.1

Tr = 2030 – 2016 = 14

G = 14 – 17.1 = -3,1 (likely)

Gq = -3,1 / 14 = -0,22

**III. Target 2030**

2010 = 5.7

2016 = 11.4

2030 = 23.0

Tn = 2030 – [2010 + ((2030 – 2010) * 11.4 – 5.7 / 23.5 – 5.7)] =

2030 – [2010 + (20 * 0,32)] = 2021 – [2010 + 6.4] = 2030 – 2016.4 = 13.6

Tr = 2030 – 2016 = 14

G = 14 – 13.6 = 0.4 (on track)

Gq = 0.4/ 14 = 0.03

**Percentage of population living within 5 km from the nearest health facility**

**I. Target 2021**

2010 = 69

2013 = 71

2021 = 85

Ad I.

Tn = 2021 – [2010 + ((2021 – 2010) * 71 – 69 / 85 – 69)] =

2021 – [2010 + (11 * 0,125)] = 2021 – [2010 + 1,375] = 2021 – 2011,375 = 9,625

Tr = 2021 – 2013 = 8

G = 8 – 9,625 = -1,63 (likely)

Gq = -1,625 / 8 = -0,20

**Health facility density per 10,000 population**

**I. Target 20121**

2015 = 1.63

2016 = 1.90

2021 = 2.00 (WHO and IP)

Tn = 2021 – [2015 + ((2021 – 2015) * 1.9 – 1.6 / 2.0 – 1.6)] =

2030 – [2015 + (6 * 0,75)] = 2021 – [2015 + 4.5] = 2021 – 2019.5 = 1.5

Tr = 2021 – 2016 = 5

G = 5 – 1.5 = +3.5 (on track)

Gq = 3.5 / 5 = 0.7

**Percentage of health facilities with all utilities ready to provide services (with water, electricity)**

**I. Target**

2015 = 55

2016 = 59

2021 = 100

Tn = 2021 – [2015 + ((2021 – 2015) * 59 – 55 / 100 – 55)] =

2021 – [2015 + (6 * 0,09)] = 2030 – [2010 + 0.53] = 2021 – 2010,5 = 10.5

Tr = 2021 – 2015 = 6

G = 6 – 10.5 = -4.5 (unlikely)

Gq = -4.5 / 6 = -0.75

**Percentage of facilities with no stock-out of tracer drugs during the period (amoxicillin, cotrimoxazole, paracetamol, ORS, iron folate, ACT, FP commodity)**

**I. Target 2021**

2011 = 62,3

2016 = 44

2021 = 95

Ad I.

Tn = 2021 – [2011 + ((2021 – 2011) * 44 – 62,3 / 95 – 62,3)] =

2021 – [2011 + (10 * -0,71)] = 2021 – [2011 -7,1] = 2021 – 2003,9 = 17,1

Tr = 2021 – 2016 = 5

G = 5 – 17,2 = -12,2 (unlikely)

Gq = -12,2 / 5 = -2,44

**OPD consultations per inhabitant per year**

**I. Target**

2010 = 0.9

2016 = 0.7

2021 = 2.0

Tn = 2021 – [2010 + ((2021 – 2010) * 0,7 – 0.9 / 2.0 – 0.9)] =

2021 – [2010 + (11 * -0,18)] = 2021 – [2010 -2.0] = 2021 – 2008 = 13

Tr = 2021 – 2016 = 5

G = 5 - 13 = -8 (unlikely)

Gq = -8 / 5 = -1.6

**II. Target**

2010 = 0.9

2016 = 0.7

2030 = 5.0 (WHO)

Tn = 2030 – [2010 + ((2030 – 2010) * 0.7 – 0.9 / 5.0 – 0.9)] =

2030 – [2010 + (20 * -0.05)] = 2030 – [2010 -1] = 2030 – 2009 = 21

Tr = 2030 – 2016 = 14

G = 14 – 21 = -7.0 (unlikely)

Gq = -7 / 14 = -0.5

**Core Health Workforce / 10,000**

**I. Target 2021**

2015 = 8,6

2016 = 11,4

2021 = 14

Ad I.

Tn = 2021 – [2015 + ((2021 – 2015) * 11,4 – 8,6 / 14,0 – 8,6)] =

2021 – [2015 + (6 * 0,52)] = 2021 – [2015 + 3,1] = 2021 – 2018,1 = 2,9

Tr = 2021 – 2016 = 5

G = 5 – 2,9 = 2,1 (on track)

Gq = 2,1 / 5 = 0,42

**Percentage of timely, accurate and complete HIS reports submitted to the MOH&SW during the year**

**I. Target 2021**

2013 = 36

2016 = 78

2021 = 90

Ad I.

Tn = 2021 – [2013 + ((2021 – 2013) * 78 – 36 / 90 – 36)] =

2021 – [2013 + (8 * 0.78)] = 2021 – [2013 + 6.2] = 2021 – 2019.2 = 1.8

Tr = 2021 – 2016 = 5

G = 5 – 1.8 = +3.2 (on track)

Gq = +3.2 / 5 = +0.64

**Per capita health expenditure per year (US$)**

**I. Target 2021**

2013 = 65

2016 = 64

2021 = 80

Ad I.

Tn = 2021 – [2013 + ((2021 – 2013) * 64 – 65 / 80 – 65)] =

2021 – [2013 + (8 * -0,07)] = 2021 – [2013 – 0,53] = 2021 – 2012,47 = 8,53

Tr = 2021 – 2016 = 5

G = 5 – 8,53 = -3,53 (unlikely)

Gq = -3,53 / 5 = -0,71

**Public expenditure for health & social welfare as percent of total public expenditure**

**I. Target 2021**

2010 = 07,8

2016 = 12,4

2021 = 15,0 (Abuja)

Ad I.

Tn = 2021 – [2010 + ((2021 – 2010) * 12,4 – 7,8 / 15 – 7,8)] =

2021 – [2010 + (11 * 0,64)] = 2021 – [2010 + 7,0] = 2021 – 2017 = 4,0

Tr = 2021 – 2016 = 5

G = 5 – 4,0 = 1,0 (on track)

Gq = 1,0 / 5 = 0,2
